# Association of neighborhood greenness with self-perceived stress, depression and anxiety symptoms in older U.S adults

**DOI:** 10.1186/s12940-018-0381-2

**Published:** 2018-04-16

**Authors:** Vivian C. Pun, Justin Manjourides, Helen H. Suh

**Affiliations:** 1 Saw Swee Hock School of Public Health, National University of Singapore, Singapore, Singapore; 2 Department of Health Sciences, Northeastern University, Boston, MA 02115 USA; 3 Department of Civil and Environmental Engineering, Tufts University, Medford, MA 02153 USA

**Keywords:** Green space, Perceived stress, Anxiety, Depression, Mediation, Effect modification

## Abstract

**Background:**

Neighborhood environment, such as green vegetation, has been shown to play a role in coping with stress and mental ill health. Yet, epidemiological evidence of the association between greenness and mental health is inconsistent.

**Methods:**

We examined whether living in green space is associated with self-perceived stress, depressive and anxiety symptoms in a nationally representative, longitudinal sample of community-dwelling older adults (*N* = 4118; aged 57–85 years) in the United States. We evaluated perceived stress, depression and anxiety symptoms using the Cohen’s Perceived Stress Scale, the Center for Epidemiological Studies – Depression, and the Hospital Anxiety and Depression Scale − anxiety subscale, respectively. Greenness was assessed for each participant using the Normalized Difference Vegetation Index at 250-m resolution, as well as a buffer of 1000-m. We conducted longitudinal analyses to assess the associations between greenness and mental health upon adjusting for confounders (e.g., education), and to examine potential mediation and effect modification.

**Results:**

An interquartile range (0.25 point) increase in contemporaneous greenness was significantly associated with 0.238 unit (95% CI: − 0.346, − 0.130) and 0.162 unit (95% CI: − 0.271, − 0.054) decrease in the perceived stress in base and multivariable models, respectively. The magnitude of the association was similar or even stronger when examining summer (− 0.161; 95% CI: − 0.295, − 0.027) and annual average of greenness (− 0.188; 95% CI: − 0.337, − 0.038), as well as greenness buffer of 1000-m. The greenness-stress association was partially mediated by physical activity (15.1% mediated), where increased greenness led to increased physical activity and less stress, and by history of respiratory diseases (− 3.8% mediated), where increased greenness led to increased respiratory disease and more stress. The association was also significantly modified by race, social support, physical function, socioeconomic status, and region. While greenness was not significantly associated with anxiety and depressive scores across all participants, significant inverse associations were found for Whites participants, and for individuals with higher socioeconomic status, who were physically active, as compared to their counterparts.

**Conclusion:**

We found a direct association of greenness with perceived stress among older adults, and an indirect association mediated through physical activity and respiratory disease history. Our study findings warrant further examination of the mediation and modification of the greenness-mental health association.

**Electronic supplementary material:**

The online version of this article (10.1186/s12940-018-0381-2) contains supplementary material, which is available to authorized users.

## Background

Mental health disorders are the third leading cause of global disease worldwide [1]. They contribute substantially to disease and disability globally and in the United States (U.S), with costs in the U.S estimated to cost US$6.0 trillion by 2030 [2]. These costs are likely to be even higher, given that mental disorders also play a critical role in the pathogenesis of cardiovascular and other chronic non-communicable diseases. Risk factors for mental health disorders are varied, including genetic profiles, socioeconomic status (SES), life circumstances, health status (e.g., body mass index), and neighborhood environment. Neighborhood environment, for example, has been shown to play a role in coping with stress and mental ill health [3, 4]. Experimental research has found strong evidence between exposure to natural environments, especially green vegetation, and recovery from physiological stress and mental fatigue, giving support to both the Stress Recovery Theory and Attention Restoration Theory [5, 6]. Consistent with this, recent European epidemiological studies have generally showed greenness/proximity to parks to be associated with lower cortisol levels and perceived stress [7], anxiety and depression [8, 9], and mental distress [10–16]. However, findings from U.S studies are non-conclusive, with conflicting associations of greenness or related proxies (e.g., greening vacant lots and vegetation, distance to parks) with stress [17–20], depressive symptoms [19, 21–23] and anxiety [8, 19, 21, 24].

To address this gap, we conducted a longitudinal analysis in the National Social Life, Health and Aging Project (NSHAP) to examine the association between greenness and self-perceived stress and depressive and anxiety symptoms, and identify significant effect modifiers that may lead to the formulation of etiologic hypotheses that advance our understanding of the pathogenetic processes involved in the greenness-mental health association. In addition, it has been suggested that green neighborhoods are aesthetically pleasing and support more diverse types of physical activity and social engagement, thereby promoting positive mental health [4, 25, 26]. Thus, we also explore whether the greenness-mental health associations are mediated by physical activity, social support, loneliness, factors that were previously found to play a role in the association between greenness and mental health [8, 26].

## Methods

### Population

NSHAP is a longitudinal, nationally representative study of community-dwelling older individuals (57–85 years), with no known cognitive impairment, living across the U.S [27]. Wave 1 recruited 3005 participants examined in 2005–2006 and Wave 2 included 3377 participants in 2011–2012, with 2261 individuals participating in both waves; 744 Wave 1 participants were either too sick to participate in Wave 2 or deceased. NSHAP over-sampled African-Americans, Latinos, men and individuals between 75 and 84 years. The overall weighted response rate was 75.5 and 76.9% for Waves 1 and 2, respectively. Participants and non-respondents did not differ with regard to their greenness measure or cognitive scores. Demographic, social, psychological, and physiological health measures were collected.

### Outcome measures

A modified Cohen’s Perceived Stress Scale (PSS-4) questionnaire, 7-item Hospital Anxiety and Depression Scale − anxiety subscale (HADS-A), and 11-item Center for Epidemiological Studies – Depression (CESD-11) Scale questionnaire were used to assess depressive and anxiety symptomatology, and self-perceived stress during the previous week, respectively [28–30]. Participants were asked to indicate their response to each item by rating the frequency of their feelings from rarely to most of the time in a 4-point Likert scale, with the higher summed scores indicating a higher perceived stress or more severe depressive or anxiety symptoms. The Cronbach’s alpha of CESD-11 and HADS-A for internal consistency was 0.80 and 0.76 for the entire NSHAP sample, respectively, whereas the reliability for PSS-4 was 0.63. Four (< 1%), 744 (12%) and 756 (12%) participants did not complete the depression, anxiety or perceived stress assessments, respectively, with missingness not related to the greenness measures. Data completeness was generally high. All missing data was imputed by maximum likelihood estimation [31]. Detailed description of the questionnaires are documented elsewhere [32].

### Exposure measure

Level of greenness surrounding each NSHAP participant’s residence was estimated using the Normalized Difference Vegetation Index (NDVI), the ratio of the difference between the near-infrared and visible (red) radiation to the sum of these two measures [33]. NDVI data were obtained from the Moderate Resolution Imaging Spectroradiometer (MODIS) from NASA’s Terra and Aqua satellites, which provide measurements every 8-days totaling 46 time-points annually, at a 250 m resolution [34]. We additionally used geographic information systems (GIS) software from ArcMap (ESRI, Redlands, CA) to estimate the contemporaneous greenness value inside radii of 1000-m buffers around each participant’s home. NDVI values range between − 1.0 and 1.0, with larger values indicating greater vegetative density [35]. We determined contemporaneous greenness for each participant using the NDVI grid point and sampling time closest to each participant’s residence and interview date, respectively. Summer and annual average greenness levels were also calculated. Three Wave 2 participants did not live in the continental U.S and thus did not have an NDVI value.

### Covariates

Loneliness was assessed using an revised 9-point University of California, Los Angeles Loneliness Scale [36]. Social support was determined by asking NSHAP participants their frequency of socializing with friends or relatives past year. Physical activity was defined by the frequency of 30-min rigorous physical activity over the past 12 months. Physical function was assessed as self-rated difficulty (ranging from no difficulty to unable to do) performing daily living activities (e.g., walking one block and across a room, using the toilet). Chronic disease conditions [e.g., diabetes, hypertension, stroke, heart failure or respiratory illnesses (i.e., emphysema, chronic obstructive pulmonary disorder, and asthma)] were assessed by asking “Has a medical doctor told you have (had) [condition]?”

Both temperature (Celsius) and PM_2.5_ concentrations (ug/m^3^) were calculated from a set of spatio-temporal generalized additive mixed models [37]. Three-day moving average exposures were estimated for temperature, while 60-day moving average was estimated for PM_2_ exposure, with exposure windows selected based on findings from previous studies [32]. Residential distance (in meters, m) to the closest U.S Census Feature Class Code A1 (primary highway with limited access), A2 (major, non-interstate highway), and A3 (secondary roads, typically state or local highway with more than two lanes) road segments was calculated as a proxy for road-traffic exposure, including traffic-related noise and air pollution [37]. Urbanicity was computed as the percentage of urban (low- and high-intensity residential, and industrial/commercial/transportation) land use within 1 km of each participant’s residence using data from the U.S Geological Survey 1992 National Land Cover Dataset [37].

### Statistical analysis

Linear mixed models were used to study the association of greenness (contemporaneous, summer and annual average), as a continuous measure, and each mental health score, accounting for repeated measurements of participants and households, and incorporating weights to account for the complex sample design in estimating standard errors for survey estimates [38]. We constructed base models adjusting for age, gender, year, season of questionnaire completion, and region. Multivariable models were constructed to additionally control for confounding from education attainment, 3-day moving average temperature, and 60-day moving average of PM_2.5_ based on the rule of thumb of 10% change in estimate. For statistically significant associations between greenness and mental health, we evaluated potential mediation by physical activity, loneliness and social support, factors that were shown to be important mediators in prior literature [8, 26]. We also examined possible mediation by covariates that were significantly associated with both greenness and mental health in our current study. Indirect effects were computed as *(coefficient of exposure in the Path Exposure− > Mediator model) × (coefficient of mediator in the Path Mediator− > Outcome model, controlling for exposure)*. Percentage of the total effect mediated was calculated as *(specific indirect effects divided by the defined total effect) × 100*, and significance of the mediating effect was assessed using Sobel test [39]. The covariates that were found to be significant mediators were not included as confounders in our multivariable models.

In addition, we further examined effect modification of the greenness-mental health associations by individual participant and neighborhood characteristics using multiplicative interaction terms. Examined characteristics included age, gender, race/ethnicity, season, region, education attainment, family income, median household income level, current smoking, physical activity, social support, history of diabetes, hypertension, stroke, heart failure or respiratory illnesses, body mass index (BMI) and physical function, loneliness, roadway distance, urbanicity. For sensitivity analyses, we restricted analyses to 1) individuals who did not currently take antidepressant medication, 2) those living in metropolitan statistical area (MSA) to evaluate the robustness of our study findings. We also re-assessed the associations using greenness measure within 1000-m buffer, as well as tertiles of greenness. All results are expressed as the mean difference in mental health scores per interquartile range (IQR; 0.25 point) increase in greenness. All analyses were performed in SAS 9.4 (Cary, NC).

## Results

Table 1 shows participant demographic, socioeconomic, health characteristics by greenness levels (see Additional file 1: Table S1 for different of measures greenness). NSHAP participants (*N* = 4118) were on average 70 years old, and nearly half were male. Participants who lived in the greenest neighborhoods tended to have lower mental health scores, to be White, more educated, physically and socially active, and live further from the main road and in an affluent neighborhood. We found from our previous study that depressive and anxiety symptoms were reversible as evidenced by the moderate correlations between measures of depression and anxiety in Wave 1 with those in Wave 2 [32]. The intra-wave correlation of perceived stress, anxiety, depressive score equaled 0.28, 0.34 and 0.55 respectively, respectively, which while statistically significant, were only moderate at best, suggesting that these symptoms may be reversible. Greenness was moderately and inversely correlated with urbanicity (*r* = − 0.47; Additional file 1: Table S2), and mildly correlated with temperature (*r* = − 0.26) and roadway distance (*r* = 0.27).

**Table 1:** Descriptive statistics (in percentage values, unless otherwise specified) of individual and neighborhood characteristics of NSHAP study participants by tertiles of greenness^a^

Characteristic	Greenness			
	3rd tertile (Greenest)	2nd tertile	1st tertile (Least green)	*p*-value
No. of participants	2106	2140	2133	
Age (year, mean ± SD)	70.2 ± 7.9	71.1 ± 8.1	71.5 ± 8.3	
Male (%)	48.9	44.8	47.1	0.0275
Race (%)				
White	82.3	72.6	58.1	< 0.0001
Black	14.8	17.3	16.6	
Hispanic non-black	1.6	8.3	21.5	
Other	1.3	1.9	3.8	
BMI (kg/m^2^, mean ± SD)	29.2 ± 6.1	29.5 ± 6.1	29.3 ± 6.2	
Current smoking (%)	13.6	14.4	14.0	0.7463
Physically active (≥1 times per week, %)	69.6	64.0	64.5	0.0001
Socioeconomic status				
Education attainment (%)				
College degree or greater	28.9	22.1	18.9	< 0.0001
High school or vocational school	55.1	57.3	54.6	
Less than high school	16.0	20.6	26.5	
Family income ($ in thousands, mean ± SD)	61.6 ± 73.1	53.2 ± 70.2	49.7 ± 70.3	
Median household income^b^ ($ in thousands, mean ± SD)	59.7 ± 28.3	54.2 ± 25.6	50.2 ± 23.9	
Diabetes (%)	22.0	23.7	21.9	0.2861
Hypertension (%)	57.4	62.1	59.2	0.0065
Stroke (%)	8.9	9.9	8.6	0.3351
Heart failure (%)	6.8	8.0	6.3	0.0726
Respiratory illness (%)	17.6	15.9	15.2	0.0951
Lonely (≥80th percentile, %)	23.4	26.6	24.4	0.0495
Social support (socializing ≥1 per month, %)	49.9	46.5	43.4	0.0001
Much difficulty get-up-and-go (≥2 score, %)	22.6	22.7	23.1	0.9160
Perceived stress score (mean ± SD)	2.2 ± 2.3	2.6 ± 2.6	2.8 ± 2.5	
Anxiety score (mean ± SD)	4.0 ± 3.4	4.3 ± 3.5	4.3 ± 3.5	
Depressive score (mean ± SD)	5.0 ± 4.8	5.4 ± 5.1	5.5 ± 5.2	
Resided in MSA (%)	88.0	85.9	88.8	0.0122
Distance to A1–A3 roadway (%)				
0–49 m	14.8	14.8	18.7	< 0.0001
50–199 m	17.0	26.3	33.3	
≥ 200 m	68.3	58.9	48.1	
Urbanicity (%, mean ± SD)	30.8 ± 26.2	52.1 ± 29.1	63.5 ± 32.0	
Temperature annual (ºC, mean ± SD)	18.5 ± 8.1	17.3 ± 8.9	16.4 ± 9.8	
PM_2.5_ annual concentration (μg/m^3^, mean ± SD)	11.4 ± 4.2	10.3 ± 4.1	9.9 ± 3.9	

^a^2,261 participants were in both wave 1 and wave 2; 744 participants were in wave 1 only; 1,113 were in wave 2 only

^b^Estimated for census tract of residence using data from the US Census Bureau (2000). Abbreviations: SD refers to standard deviation; BMI refers to body mass index; NDVI refers to the normalized difference vegetation index; MSA refers to metropolitan statistical area; PM_2.5_ refers to particulate matter with aerodynamic diameter of 2.5 micro or less

### Perceived stress

**Fig. 1:**
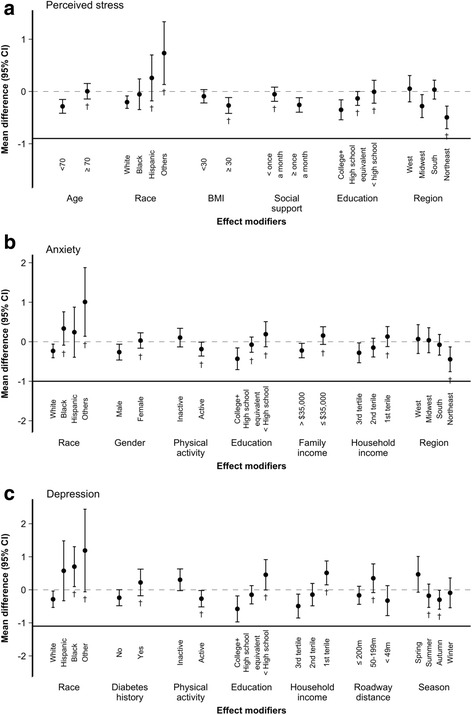
Mean difference (95% CI) for increment in contemporaneous greenness (at 250-m buffer) and **a**) perceived stress, **b**) anxiety symptoms, and **c**) depressive symptoms among NSHAP participants, stratified by effect modifiers in multivariable models^1,2^. ^1^ Multivariable models adjusted for age, gender, questionnaire year and season, region, education attainment, 3-day moving average of temperature and 60-months moving average of PM_2.5_. ^2^ First category of each modifier as reference group; daggers represent statistically significant effect modification (p-interact < 0.05)

An IQR increase in contemporaneous greenness, modeled as a continuous measure, was significantly associated with 0.238 unit (95% CI: − 0.346, − 0.130) decrease in perceived stress score in base models (Table 2). The association was attenuated but remained statistically significant (− 0.162; 95% CI: − 0.346, − 0.130) in multivariable models adjusting for education, temperature and PM_2.5_. The magnitude of the association was similar or even stronger when summer (− 0.161; 95% CI: − 0.295, − 0.027) and annual greenness average (− 0.188; 95% CI: − 0.337, − 0.038) were examined. Sensitivity analyses restricting to participants who did not currently take antidepressant medication or those living in MSAs, as well as analyses using greenness at 1000-m buffer, and tertiles of greenness, respectively, show consistent results with those from the primary multivariable models (Additional file 1: Table S3 − S5). The inverse association between greenness and stress was statistically significant among participants who aged < 70, White, had higher BMI, those who were more socially active, had at least college education, or lived in the Northeastern U.S (p_interact_ < 0.05; Fig. 1). No statistically significant differences in the association were found by gender, lifestyle habits, disease or health conditions, and neighborhood characteristics.

**Table 2:** Difference (95% CI) in symptoms of mental ill health associated with an interquartile-range increase in greenness at 250-m buffer zone

Greenness	Perceived Stress	Anxiety	Depression
*Contemporaneous*			
Base model ^a^	− 0.238 (− 0.346, − 0.130)^**^	− 0.178 (− 0.333, − 0.022)^**^	− 0.299 (− 0.524, − 0.074)^**^
Multivariable model ^b^	− 0.162 (− 0.271, − 0.054)^**^	− 0.110 (− 0.266, 0.047)	− 0.150 (− 0.374, 0.075)
*Summer average*			
Base model ^a^	− 0.270 (− 0.400, − 0.139)^**^	−0.167 (− 0.355, 0.022)^*^	− 0.529 (− 0.809, − 0.249)^**^
Multivariable model ^b^	− 0.161 (− 0.295, − 0.027)^**^	−0.080 (− 0.275, 0.115)	− 0.232 (− 0.519, 0.055)
*Annual average*			
Base model ^a^	− 0.319 (− 0.465, − 0.172)^**^	−0.218 (− 0.430, − 0.005)^**^	−0.616 (− 0.933, − 0.300)^**^
Multivariable model ^b^	− 0.188 (− 0.337, − 0.038)^**^	−0.104 (− 0.322, 0.115)	− 0.274 (− 0.596, 0.048)^*^

^a^Base models adjusted for age, gender, questionnaire year and season, and region

^b^Multivariable model adjusted for age, gender, questionnaire year and season, region, education attainment, 3-day moving average of temperature and 60-months moving average of PM_2.5_

* *P* < 0.10; ** *P* < 0.05

In mediation analyses of the association between contemporaneous greenness and perceived stress, greenness was found to indirectly associated with perceived stress through physical activity (15.1%) (Table 3), with increasing greenness positively related to physical activity, which was in turn associated with decreased stress. To a lesser extent, the greenness-stress association was also partially mediated by history of respiratory disease (**−** 3.8%), with greater greenness linked to greater reporting of respiratory diseases history such as emphysema, chronic obstructive pulmonary disorder, and asthma, which in turn results in higher self-perceived stress.

**Table 3:** Mediation analysis of the association between contemporaneous greenness (at 250-m buffer) and perceived stress^a^

Mediator	Path E➔O (direct effect)	Path E➔M	Path M➔O	Path E➔M➔O (indirect effect)	Percent mediated^b^
History of respiratory diseases	− 0.169 (0.055)^**^	0.021 (0.009)^**^	0.295 (0.081)^**^	0.006 (0.003)^**^	**−3.80%**
Physical activity	−0.138 (0.055)^**^	0.124 (0.035)^**^	−0.197 (0.020)^**^	−0.025 (0.007)^**^	**15.12%**
Social support	−0.155 (0.055)^**^	0.037 (0.028)	−0.190 (0.025)^**^	−0.007 (0.005)	4.37%
Loneliness	−0.146 (0.053)^**^	−0.050 (0.042)	0.319 (0.016)^**^	−0.016 (0.014)	9.93%

^a^The total effect of the association between greenness and perceived stress was −0.162 (−0.271, −0.054). Both direct and indirect effect was expressed as the mean difference of the perceived stress per 0.25 unit increment in greenness, in the primary multivariable models adjusted for age, gender, questionnaire year and season, and region, education attainment, 3-day moving average of temperature and 60-months moving average of PM_2.5_. * *p*<0.10; ** *p*<0.05.

^b^Bold texts refer to the statistically significant mediation effect according to Sobel test (p_indirect_ <0.05)

Abbreviations: *E* refers to exposure (i.e., greenness), *O* refers to outcome (i.e., perceived stress), *M* refers to mediator

### Anxiety

Increasing contemporaneous greenness was significantly associated with reduced anxiety symptoms in base models (− 0.178; 95% CI: − 0.333, − 0.022; Table 2). The association, however, became insignificant upon adjusting for additional confounders (− 0.110; 95% CI: − 0.266, 0.047). Similar pattern of associations was observed for summer and annual greenness average. Nonetheless, White and male participants, and those who were physically active, or had higher socioeconomic status (e.g., family income) had lower anxiety scores associated with increased greenness as compared to their counterparts (Fig. 1). Moreover, the inverse association between greenness and anxiety was statistically significant and in Northeastern U.S.

### Depression

Similarly, greenness, irrespective of duration measures, was associated with depressive symptoms in base model (− 0.299; 95% CI: − 0.524, − 0.074 for contemporaneous greenness), but the association diminished in the multivariable models (Table 2). While no modification of the greenness-mental illness health associations by most individual and neighborhood characteristics was observed, significant effect modification was found in which White participants, and those with no history of diabetes, physically active, had higher socioeconomic status had lower depressive scores associated with increased greenness, in comparison to their counterparts (Fig. 1). Mediation was not evaluated given the null associations between greenness and anxiety/depressive scores across all participants.

## Discussion

**Fig. 2:**
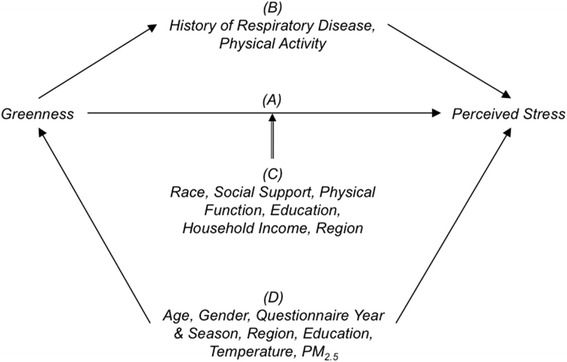
DAG demonstrating the relationship among contemporaneous greenness, perceived stress and their mediators, confounders, and effect modifiers. Pathway **a** represents of the direct, unmediated, association between greenness and perceived stress. Pathway **b** demonstrates the role of mediators, in which greenness has an indirect effect of which respiratory disease history and physical activity are on the causal pathway. Pathway **c** shows the effect modifiers that modify the greenness’ effect on the perceived stress. Pathway **d** depicts the presence of confounders because they predict both the greenness and stress

In our nationally representative sample of U.S community-dwelling older adults, we evaluated whether greenness was associated with three short-term mental ill health indictors. We found that increased greenness was significantly associated with lower self-perceived stress during the past week, upon controlling for confounders. We also found partial mediation of the greenness-stress association by physical activity and history of respiratory diseases. Associations were modified by several individual-specific characteristics, with the significantly inverse association among participants who were relatively young, White, well-educated, more socially active, and those had higher BMI or lived in the Northeast. Although greenness was not significantly associated with anxiety and depression across all participants, our results show that greenness was associated with larger reductions in anxiety and depression scores in Whites, individuals who had higher SES, and were physically active, as compared to their counterparts. A directed acyclic graph (DAG) was shown in Fig. 2 to illustrate the role of confounders, mediators, and modifiers in the association between greenness and perceived stress.

Our findings of inverse association of greenness with perceived stress overall, and with anxiety/depression for certain subgroups are generally consistent with previous longitudinal studies of greenness and mental distress, a global measure of mental health [9–13] and cross-sectional studies on self-reported stress [7, 40]. For instance, three studies of the British Household Panel Survey found significantly lower mental distress associated with living in greener urban areas in England [10–12], while a Swedish study found no link between access to greenness and mental distress [13], perhaps due to its use of a relatively crude exposure measure (e.g., serene, lush) that may not capture subtle differences in greenness as was possible using the NDVI. Our observed protective effect of greenness on mental health is also in line with a British study that showed that pregnant women living in the greener quintiles were 18–23% less likely to report depressive symptoms than those living in the least green quintile [9]. While supportive, the relevance of findings from these European studies to our study may be limited, given differences in housing and land use [41].

Comparison of our findings to those from U.S studies, however, are limited, as the only U.S studies to date are cross-sectional studies conducted in cities or other localized geographic regions [18, 19, 21], with mixed results. Our results for perceived stress are most consistent with that from Fan et al. [18], who showed a significant mitigation of perceived stress (log scale, β = − 0.044) associated with neighborhood greenness in a probability sample of Chicago residents, and Beyer et al. [21], who reported significant beneficial effect of greenness (i.e., tree canopy & NDVI combined) on perceived stress (β = − 0.735) among Wisconsin residents. Our study, however, differs from a recent Seattle, Washington study of 4338 monozygotic twins that found no association between NDVI and perceived stress upon adjusting for income and physical activity [19]. This discrepancy may result from our substantially different study populations, as the twin population consisted of predominately White middle-aged women, whereas our study population consisted of older, more ethnically diverse men and women living across the U.S Notably, we found region of the country to modify our greenness-stress association, with this association being significant and strongest in the Northeastern U.S, but not in other regions including the West, where the majority of the twins in the Seattle study resided. Strong beneficial effects of greenness on stress for participants in the Northeast may be attributed to the Northeast region’s high population and housing density and large proportion of urban land [42, 43]. As a result, small changes in the level greenness may have greater effects on mental health. Further research should aim to explore possible regional differences in more detail.

Similarly, more research is needed to support our finding of effect modification by SES, where we showed the significant inverse association between greenness and perceived stress only for individuals with higher education. This finding is counter to prevailing theory that the beneficial effect of greenness is stronger among individuals with lower education or SES due to the fact that these individuals spend more time near their homes and thus use and interact more frequently with their immediate surrounding environment [9, 15]. However, it is possible that the key modifying factor is not SES but rather time spent near home, where children, housewives, and elderly (e.g., community-dwelling older adults assessed in our study) may spend more time near their homes as compared to the other populations [e.g., pregnant women [9]] examined in previous studies [15]. Our finding on the modifying effect of BMI lends support to our argument, in which obese individuals tended to stay around their residence than those with lower BMI, thus interact more with their surrounding greenness.

Notably, our findings that show greenness to be associated with lower stress levels through increased physical activity are consistent with theories that relate green vegetation in the natural environment with more positive emotional states, physiological activity levels, and behavior and cognitive functioning [5, 44]. Several mechanisms through which greenness influences mental health have been postulated, including the enhancement of health and well-being [15], promotion of physical activity [9, 45], provision of healthy environment and better air quality [46], and facilitation of social contact [8]. While consistent with prevailing hypotheses and some recent literature [7, 9, 40, 47, 48], our findings are in contrast with other studies that found little or no mediation by physical activity [18, 40, 49]. Possible explanations for the conflicting findings include difference in geographic area coverage, where more localized geographic area (e.g., Chicago) may result in lower variation in exposure in some studies [18, 40], and in the examination of recreational walking in the English study as compared to rigorous physical activity (e.g., sports, exercise classes or heavy housework) in the current study [49]. Further, although we did not find significant positive mediation by social support, we did find socially active individuals to have lower perceived stress associated with greenness as compared to their socially inactive counterparts, which is consistent with prevailing hypotheses [50]. It is possible that the insignificance of social support as a mediator may result from our measure of social support, which was relatively crude as compared to the Social Support List, a modified 19-items questionnaire with good construct validity and high reliability, that was used in previous study that reported partial mediation of the relation between green space and health by social contact [26]. Alternatively, our findings may reflect differing impacts of social support on greenness and mental health, as results from a Chicago study provided yet a different relation of social support, greenness, and mental health, finding social support to counteract the beneficial effect of neighborhood greenness on perceived stress among Black and Hispanic adults. The authors suggested that the high levels of park accessibility in the Chicago neighborhoods may obscure their study findings [18].

In addition to physical activity, we observed history of respiratory diseases to partially mediate the association of decreasing greenness and increased stress, although the indirect effect was small. Contrary to the common belief that greenness should have a beneficial effect on health, we found greenness to be positively associated with history of respiratory disease. Our finding is supported by previous studies that showed that greenness was associated with increased respiratory diseases (e.g., asthma), possibly reflecting the mediating influence of pollen and other aeroallergens on this association [51, 52]. Our positive associations between greenness and respiratory disease may further reflect the fact that over 90% of our cohort resided in MSAs, with most not moving during our study period, given results from several studies showing that associations between greenness and respiratory conditions tend to be positive for populations living in urban areas [53–55]. Fuertes et al. [55], for example, found that green space measured by NDVI was inversely associated with allergic rhinitis and eye and nose symptoms among children living in rural areas, but was positive associated among children living in urban areas. Alternatively, it may also be possible that individuals with emphysema or COPD may tend to move away from urbanized (polluted) environments to greener (less-polluted) areas; however, this hypothesis has not yet been examined.

As with perceived stress, we found greenness to be associated with decreased anxiety and depressive symptoms in specific segments of our population, consistent with existing hypotheses that anxiety and depression are influenced by the amount of neighborhood greenness [8, 9, 19, 24]. We found greater greenness to be associated with lower depression and anxiety scores only in men, which is similar to a British longitudinal study reporting associations between improved mental distress and greenness among men, with only moderate associations among older women [11]. We observed greenness to be significantly associated with reduced anxiety/depression symptoms among White but not Black and Hispanic participants, and among individuals with higher SES or living in higher SES neighborhoods. Furthermore, we found the effect modification by physical activity, in which greater greenness was associated with improved anxiety and depression symptoms only among individuals who were physically active. These findings are similar with previous evidence showing that the association between greenness and depressive symptoms was significant for individuals who were active [9], and also with collective evidence from literature review that the natural environment increases the positive effects of physical activity on well-being [45].

Our study has several limitations. First, CESD and HADS-A are not clinical diagnostic instruments, nor are they designed to assess chronic mental disorder. However, these questionnaires are widely used screening tools for current depressive and anxiety symptom severity in the somatic, psychiatric and general population settings [56]. Likewise, high PSS has been shown to correlate with high serum cortisol, a stress biomarker [57]. Second, exposure misclassification remains a concern in environmental epidemiology. While NDVI is a valid measure of greenness [58], it cannot distinguish between different types of green area such as tree canopy or parks, which may influence mental health through different mechanisms [59]. Third, we could not rule out the likelihood of self-selection bias in that participants with higher SES being more likely to select living in greener neighborhoods and to have lower mental health scores. Lastly, findings from the current study may not be generalizable to younger age groups.

Despite these limitations, our study is the first to examine the association of neighborhood greenness and mental health in a nationally representative sample of older, community-dwelling Americans. The longitudinal nature of our study design is a major strength, as previous U.S studies were cross-sectional design. We evaluated two affective and one psychological measures of mental health to provide a comprehensive picture of effect of greenness on mental health. We were also one of the first to explore how the greenness-mental health associations might be modified or mediated by a host of individual and/or neighborhood characteristics, and we are not aware of previous published results in older adults. In addition, our study was well-powered to detect meaningful associations and adjusted for confounding from individual- and census-level SES, air pollution and weather.

## Conclusions

We observed direct association of neighborhood greenness with perceived stress in a nationally representative sample of U.S older adults, and indirect association through pathways related to physical activity and respiratory disease history. Individuals with higher SES and those who were active were more likely to have better mental health scores associated with increased greenness. Our study findings warrant prospective studies to further examine the mediation and modification of the link between greenness and mental health.

## Additional file

Additional file 1: **Table S1.** Descriptive statistics of the greenness measures. **Table S2.** Pearson correlations of neighborhood variables. **Table S3.** Mean difference (95% CI) in self-perceived stress, anxiety and depression symptoms associated with an interquartile-range increase in greenness. **Table S4.** Difference (95% CI) in symptoms of mental ill health associated with an interquartile-range increase in contemporaneous greenness in 1,000-m buffer zones. **Table S5.** Mean difference (95% CI) in self-perceived stress, anxiety and depression symptoms associated with tertiles of greenness. (DOCX 33 kb)

## Abbreviations

CESD-11: 11-item Center for Epidemiological Studies – Depression
HADS-A 7: item Hospital Anxiety and Depression Scale − anxiety subscale
MODIS: Moderate Resolution Imaging Spectroradiometer
MSA: Metropolitan statistical area
NASA: The National Aeronautics and Space Administration
NDVI: Normalized Difference Vegetation Index
NSHAP: National Social Life, Health and Aging Project
PM2.5: Particulate matter with aerodynamic diameter of 2.5 μm or less
PSS-4: 4-item Cohen’s Perceived Stress Scale
SES: Socioeconomic status
U.S: United States
USD: United States Dollar
