# Supplementary material for: Association of neighborhood greenness with self-perceived stress, depression and anxiety symptoms in older U.S adults
Source: Environ Health. 2018 Apr 16;17:39. doi: 10.1186/s12940-018-0381-2 (PMC5902952; doi:10.1186/s12940-018-0381-2)
Supplement: Supplementary file 1 — Table S1. Descriptive statistics of the greenness measures. Table S2. Pearson correlations of neighborhood variables. Table S3. Mean difference (95% CI) in self-perceived stress, anxiety and depression symptoms associated with an interquartile-range increase in greenness. Table S4. Difference (95% CI) in symptoms of mental ill health associated with an interquartile-range increase in contemporaneous greenness in 1,000-m buffer zones. Table S5. Mean difference (95% CI) in self-perceived stress, anxiety and depression symptoms associated with tertiles of greenness. (DOCX 33 kb) [file 12940_2018_381_MOESM1_ESM.docx]

**Additional file 1**

**Table S1.** Descriptive statistics of the greenness measures.

| **Greenness** | **Minimum** | **Mean** | **Maximum** | **IQR** |
| --- | --- | --- | --- | --- |
| Contemporaneous measure at 250-m buffer | 0.00 | 0.54 | 0.91 | 0.25 |
| Summer average  at 250-m buffer | 0.04 | 0.56 | 0.84 | 0.22 |
| Annual average  at 250-m buffer | 0.02 | 0.50 | 0.76 | 0.18 |
| Contemporaneous measure at 1,000-m buffer | 0.07 | 0.51 | 0.90 | 0.25 |

**Table S2.** Pearson correlations of neighborhood variables.

|  | Greenness | Household income | Distance to roadway | Urbanicity | PM_2.5_ | Temperature |
| --- | --- | --- | --- | --- | --- | --- |
| Greenness | 1.0 |  |  |  |  |  |
| Household income | 0.19** | 1.0 |  |  |  |  |
| Distance to roadway | 0.27** | 0.10** | 1.0 |  |  |  |
| Urbanicity | −0.47** | −0.15** | −0.41** | 1.0 |  |  |
| PM_2.5_ | 0.06** | −0.17** | −0.04** | 0.32** | 1.0 |  |
| Temperature | −0.26** | −0.25** | 0.04** | 0.01 | −0.02* | 1.0 |
| * P<0.10; ** P<0.05 | | | | | | |

**Table S3.** Mean difference (95% CI) in self-perceived stress, anxiety and depression symptoms associated with an interquartile-range increase in greenness.

|  | | **Perceived Stress** | **Anxiety** | **Depression** |
| --- | --- | --- | --- | --- |
| Multivariable model ^a^ | −0.162 (−0.271, −0.054)** | | −0.110 (−0.266, 0.047) | −0.150 (−0.374, 0.075) |
| MV model restricting to those with no antidepressant medication | −0.146 (−0.260, −0.032)** | | −0.140 (−0.303, 0.023) | −0.139 (−0.373, 0.095) |
| MV model restricting to those living to MSA | −0.177 (−0.294, −0.059)** | | −0.136 (−0.306, 0.033) | −0.224 (−0.473, 0.025) |
| ^a^ Multivariable (MV) model adjusted for age, gender, questionnaire year and season, region, education attainment, 3-day moving average of temperature and 60-months moving average of PM_2.5_.  * P<0.10; ** P<0.05 | | | | |

**Table S4.** Difference (95% CI) in symptoms of mental ill health associated with an interquartile-range increase in contemporaneous greenness in 1,000-m buffer zones.

| **Greenness** | **Perceived Stress** | **Anxiety** | **Depression** |
| --- | --- | --- | --- |
| ***Primary measure (250-m)*** |  |  |  |
| Base model ^a^ | −0.238 (−0.346, −0.130)** | −0.178 (−0.333, −0.022)** | −0.299 (−0.524, −0.074)** |
| Multivariable model ^b^ | −0.162 (−0.271, −0.054)** | −0.110 (−0.266, 0.047) | −0.150 (−0.374, 0.075) |
| ***1,000-m buffer*** |  |  |  |
| Base model ^a^ | −0.245 (−0.369, −0.121)** | −0.153 (−0.331, 0.026) | −0.414 (−0.674, −0.154)** |
| Multivariable model ^b^ | −0.163 (−0.287, −0.038)** | −0.070 (−0.251, 0.110) | −0.293 (−0.555, −0.031)** |
| ^a^ Base models adjusted for age, gender, questionnaire year and season, and region.  ^b^ Multivariable model adjusted for age, gender, questionnaire year and season, region, education attainment, 3-day moving average of temperature and 60-months moving average of PM_2.5_.  * P<0.10; ** P<0.05 | | | |

**Table S5.** Mean difference (95% CI) in self-perceived stress, anxiety and depression symptoms associated with tertiles of greenness.

|  | **Perceived Stress** | **Anxiety** | **Depression** |
| --- | --- | --- | --- |
| **Base model^a^** |  |  |  |
| *Greenness* |  |  |  |
| 3rd terile (greenest) | −0.375 (−0.544, −0.206)** | −0.165 (−0.407, 0.077) | −0.251 (−0.598, 0.096) |
| 2nd terile | −0.153 (−0.310, 0.004)* | 0.089 (−0.135, 0.313) | −0.025 (−0.343, 0.292) |
| 1st tertile (least green) | 1.00 | 1.00 | 1.00 |
| *p-trend* | *<0.0001* | *0.1253* | *0.1321* |
| **Multivariable model^b^** |  |  |  |
| *Greenness* |  |  |  |
| 3rd terile (greenest) | −0.267 (−0.436, −0.098)** | −0.065 (−0.309, 0.178) | −0.044 (−0.391, 0.303) |
| 2nd terile | −0.082 (−0.238, 0.075) | 0.161 (−0.063, 0.386) | 0.124 (−0.192, 0.441) |
| 1st tertile (least green) | 1.00 | 1.00 | 1.00 |
| *p-trend* | *0.0014* | *0.4537* | *0.7118* |
| ^a^ Base models adjusted for age, gender, questionnaire year and season, region.  ^b^ Multivariable model adjusted for age, gender, questionnaire year and season, region, education attainment, 3-day moving average of temperature and 60-months moving average of PM_2.5_.  * P<0.10; ** P<0.05 | | | |
